# Properties of virion transactivator proteins encoded by primate cytomegaloviruses

**DOI:** 10.1186/1743-422X-6-65

**Published:** 2009-05-27

**Authors:** Iain P Nicholson, Jane S Sutherland, Tanya N Chaudry, Earl L Blewett, Peter A Barry, Mary Jane Nicholl, Chris M Preston

**Affiliations:** 1 Medical Research Council Virology Unit, Church Street, Glasgow G11 5JR, UK; 2 Department of Biochemistry and Microbiology, Oklahoma Center for Health Sciences College of Osteopathic Medicine, Oklahoma State University, 1111 West 17th Street, Tulsa, Oklahoma 74107-1898, USA; 3 Center for Comparative Medicine, Department of Pathology and Laboratory Medicine, California National Primate Research Center, University of California, Davis, Davis, California 95616, USA

## Abstract

**Background:**

Human cytomegalovirus (HCMV) is a betaherpesvirus that causes severe disease in situations where the immune system is immature or compromised. HCMV immediate early (IE) gene expression is stimulated by the virion phosphoprotein pp71, encoded by open reading frame (ORF) UL82, and this transactivation activity is important for the efficient initiation of viral replication. It is currently recognized that pp71 acts to overcome cellular intrinsic defences that otherwise block viral IE gene expression, and that interactions of pp71 with the cell proteins Daxx and ATRX are important for this function. A further property of pp71 is the ability to enable prolonged gene expression from quiescent herpes simplex virus type 1 (HSV-1) genomes. Non-human primate cytomegaloviruses encode homologs of pp71, but there is currently no published information that addresses their effects on gene expression and modes of action.

**Results:**

The UL82 homolog encoded by simian cytomegalovirus (SCMV), strain Colburn, was identified and cloned. This ORF, named S82, was cloned into an HSV-1 vector, as were those from baboon, rhesus monkey and chimpanzee cytomegaloviruses. The use of an HSV-1 vector enabled expression of the UL82 homologs in a range of cell types, and permitted investigation of their abilities to direct prolonged gene expression from quiescent genomes. The results show that all UL82 homologs activate gene expression, and that neither host cell type nor promoter target sequence has major effects on these activities. Surprisingly, the UL82 proteins specified by non-human primate cytomegaloviruses, unlike pp71, did not direct long term expression from quiescent HSV-1 genomes. In addition, significant differences were observed in the intranuclear localization of the UL82 homologs, and in their effects on Daxx. Strikingly, S82 mediated the release of Daxx from nuclear domain 10 substructures much more rapidly than pp71 or the other proteins tested. All UL82 homologs stimulated the early release of ATRX from nuclear domain 10.

**Conclusion:**

All of the UL82 homolog proteins analysed activated gene expression, but surprising differences in other aspects of their properties were revealed. The results provide new information on early events in infection with cytomegaloviruses.

## Background

Human cytomegalovirus (HCMV) is an important human pathogen that causes fetal damage and organ transplant rejection, and additionally represents a serious problem in immunocompromised individuals such as AIDS patients. The predicted 165 HCMV-encoded genes are expressed in a regulated program in which immediate early (IE) proteins are produced prior to early and late gene products. A complex promoter/enhancer element, the major immediate early promoter (MIEP), controls transcription of the most abundantly expressed IE locus, while the two major proteins specified by this locus modulate all classes of viral gene expression through positive and negative mechanisms. Proteins that enter the cell as components of the infecting particle provide an additional level of transcriptional regulation, and one of the most extensively studied of these, the tegument phosphoprotein pp71 (encoded by HCMV gene UL82) activates expression from the MIEP in a variety of assay systems [1-5]. HCMV mutants lacking the UL82 coding region are impaired for viral gene expression, especially after infection at low multiplicity, demonstrating that pp71 is important for virus replication in permissive cells [6,7].

It has recently become clear that pp71 exerts its positive effect on IE gene expression by counteracting an intrinsic antiviral defence that is dependent on the cell protein Daxx. This protein is found in the cytoplasm, where it regulates apoptosis [8,9], and also in nuclear structures known as nuclear domain 10 (ND10) [10-12]. Within the nucleus, Daxx functions as a repressor of gene expression, primarily by localizing chromatin-associated inhibitory factors, such as histone deacetylases, to relevant promoter regions [13-15]. Upon HCMV infection, pp71 migrates to the nucleus where it localizes to ND10 by binding to Daxx [4,16]. The outcome of this interaction is the neutralization of repression by Daxx and consequent efficient transcription of viral IE genes [15,17-20]. Recent studies indicate that pp71 stimulates the release of ATRX, a cell protein with chromatin remodelling activity, from ND10 in a very early event after infection, suggesting that ATRX is an important component of the cellular intrinsic defences to HCMV [21].

Many studies have determined the activity of pp71 by transfection of plasmids encoding the protein together with a reporter plasmid [1,2,22,23]. As an alternative method, we have used herpes simplex virus type 1 (HSV-1) mutants as vectors to enable expression of pp71. The basic vector, *in*1312, has mutations that inactivate the transcriptional functions of three crucial HSV-1 proteins. The changes are an insertion in the coding region of the virion component VP16, deletion of the RING domain of the IE protein ICP0 and introduction of a temperature sensitive mutation in the essential transactivator ICP4. As a consequence of these mutations, *in*1312 is impaired for transcription of its own genome and can be used as a vector for the expression of transgenes in a range of cell types that are permissive for HSV-1. Infection with *in*1312 derivatives that express pp71 is an efficient means of introducing the protein into cells. The activity of pp71 can be assayed by superinfection with a second *in*1312 derivative that contains a reporter gene, usually *E. coli lacZ*, controlled by various promoters. This system has been used to demonstrate that pp71 activates expression from a range of viral and cellular promoters in human, monkey and mouse cell lines, and also in mouse neurons *in vivo *[3,5].

Further experiments with *in*1312 derivatives that express both β-galactosidase and pp71 revealed an additional property of pp71, namely that it counteracts repression of the HSV-1 genome. After infection of human fibroblasts, *in*1312 is competent for transcription for only a few hours, after which it becomes repressed by cellular mechanisms. Once repressed, the genome remains in a stable 'quiescent' state for more than 12 days in cell cultures. If the infecting *in*1312 derivative expresses pp71, however, a gradual reversal of the quiescent state is observed, signified by production of β-galactosidase many days after initial infection in cultures maintained at 38.5°C, and by resumption of virus replication in cultures downshifted to 32°C to reverse the temperature sensitive defect of ICP4 [24].

Although pp71 has been studied extensively, there have been few reports that address the functional activities of homologs encoded by other cytomegaloviruses, and none that describe the properties of UL82 homologs specified by non-human primate cytomegaloviruses. In murine cytomegalovirus (MCMV), three open reading frames (ORFs) in the 'UL82 family' (M82, M83 and M84) exist where there are only two in the case of HCMV (UL82 and UL83), and it has been suggested that gene duplication resulted in an MCMV UL82-like ancestor that gave rise to both M82 and M83 [25]. MCMV mutants lacking M83 replicated normally in tissue culture, suggesting that M83 is not an activator of IE gene expression [26]. Further studies concluded that neither M82 nor M83 interacts with Daxx, and therefore it is unclear whether MCMV encodes a virion protein that is functionally analogous to pp71 [27]. The UL82 homolog encoded by guinea pig cytomegalovirus (GPCMV) stimulated the transfection efficiency of GPCMV DNA and complemented the replication of a HSV-1 VP16 mutant. Furthermore, a GPCMV UL82-null mutant was impaired for replication in culture. Taken together, these observations imply that the GPCMV UL82 gene product is, like pp71, an activator of gene expression [28,29].

In the studies presented here, we compared the properties of pp71 with homologs encoded by non-human primate cytomegaloviruses in three ways. The ability to function as activators of gene expression was tested, the capacity to overcome repression of HSV-1 genomes was investigated, and the intranuclear distributions of the proteins, were determined. The results reveal that, despite the overall similarity of the UL82 homologs, there are surprising differences in their properties.

## Results

**Figure 1:**
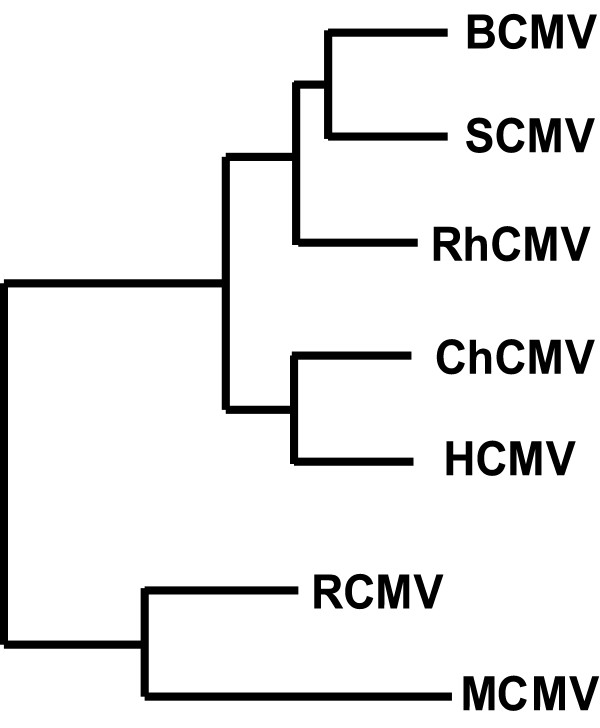
**Phylogenetic tree showing the relationships of UL82 homologs**. A neighbour-joining tree was derived from the aligned amino acid sequences, and midpoint rooted.

Experiments were initially carried out to identify and clone the simian CMV (SCMV) homolog of pp71. Through a process of 'chromosome walking', an ORF at the appropriate genome location was identified and sequenced [Genbank: FJ610688]. The predicted amino acid sequence was closely similar to that of pp71, and the gene was named S82. The sequence was confirmed in a project to determine the entire nucleotide sequence of SCMV (A. Dolan, personal communication). An evolutionary tree was constructed to include the primate UL82 homologs, together with the rat CMV UL82 homolog and MCMV M82 ORF (Fig. 1). S82 was most closely related to the baboon CMV (BCMV) protein B82, and these two sequences plus the rhesus CMV (RhCMV) protein Rh82 formed a branch separate from that which included the chimpanzee CMV (ChCMV) protein UL82 and HCMV pp71. The relationships between the proteins, apart from S82, are as published previously [28]. As expected, in view of its close similarity to other primate homologs, S82 contained sequence motifs thought to be functionally important, including two Daxx-interaction domains [4,7], a retinoblastoma protein binding motif [30,31] and, as reported previously, an internal region named the dUTPase-related domain [32].

To investigate the functional activity of S82, an N-terminal YFP tag was fused to the ORF and the hybrid construct, controlled by the HCMV MIEP, cloned into the HSV-1 mutant *in*1312 (Table 1). The resulting virus, *in*1305, was compared with *in*1316, an analogous recombinant that expresses YFPpp71 instead of YFPS82 [24], using a functional assay described previously [3,5]. Cells were infected with *in*1305 or *in*1316, and after incubation for 3 h, superinfected with a second *in*1312 derivative containing the *E. coli lacZ *coding region controlled by various promoters, as a reporter virus. Cultures were harvested after a further 5 h and β-galactosidase assays performed. To investigate possible cell type or promoter specificity, the experiments were performed in human (HFFF2), African green monkey (Vero) or mouse (3T3) cells, with a range of promoters driving β-galactosidase expression in the reporter virus (Table 2).

**Table 1:** HSV-1 *in*1312-based recombinants used in the study^1^

Mutant	Transgenes expressed^2^
*in*1312	None
*in*1374	HCMV IE-lacZ (UL43)
*in*1316	YFPpp71 (TK)
*in*1305	YFPS82 (TK)
*in*1310	YFPpp71 (TK), HCMV IE-lacZ (UL43)
*in*0150	YFPS82 (TK), HCMV IE-lacZ (UL43)
*in*0144	YFPRh82 (TK), HCMV IE-lacZ (UL43)
*in*0145	YFPB82 (TK), HCMV IE-lacZ (UL43)
*in*0146	YFPCh82 (TK), HCMV IE-lacZ (UL43)
*in*1357	SCMV IE-lacZ (TK)
*in*1382	HCMV IE-lacZ (TK)
*in*1383	HSV ICP0-lacZ (TK)
*in*1372	HCMV IE-Cre (TK)

1. The parental mutant, *in*1312, has mutations that inactivate the transcriptional activities of HSV-1 proteins VP16, ICP0 and ICP4. The construction of the mutants is described in materials and methods.

2. The insertion site in the *in*1312 genome is shown in brackets.

**Table 2:** Stimulation of gene expression by pp71 and S82^1^

Cells	Preinfection	Stimulation of β-galactosidase expression^2^		
		*in*1382	*in*1357	*in*1383
HFFF2	*in*1372	0.7	0.9	ND
HFFF2	*in*1316	4.8	2.7	ND
HFFF2	*in*1305	4.8	3.3	ND
				
Vero	*in*1372	0.6	ND	ND
Vero	*in*1316	5.1	4.7	ND
Vero	*in*1305	3.7	4.0	ND
				
3T3	*in*1316	12.6	7.3	5.6
3T3	*in*1305	10.8	6.6	4.3

1. Monolayers were mock infected or infected with *in*1372 (a control virus that expresses Cre recombinase), *in*1316 (expresses pp71) or *in*1305 (expresses S82) at moi 2, incubated at 38.5°C for 3 h, and infected with a second reporter virus (moi 0.5) that contained the *lacZ *coding region controled by the HCMV MIEP (*in*1382), the SCMV MIEP (*in*1357) or the HSV-1 ICP0 IE promoter (*in*1383). After incubation for a further 5 h at 38.5°C, cell extracts were analysed for β-galactosidase activity.

2. The stimulation of β-galactosidase expression compared with the value for extracts of cells mock infected prior to infection with the reporter virus. Backgound activity from cells mock infected throughout was subtracted from all values. The means from at least two independent determinations are presented.

In all cell types tested, preinfection with *in*1305 resulted in stimulation of β-galactosidase production, demonstrating that S82 is an activator of gene expression. The activity of S82 was comparable to that of pp71 in human, African green monkey and mouse cells, irrespective of the promoter in the reporter virus. There was no preference for the homologous promoter, as preinfection with *in*1305 resulted in an equivalent stimulation of expression when either the HCMV or the SCMV MIEP elements controlled *lacZ*. Similarly, pp71 expressed by preinfection with *in*1316 stimulated expression from the HCMV and SCMV MIEPs to similar extents. In addition, the use of a homologous host cell did not influence the relative degree of stimulation, since *in*1305 and *in*1316 exhibited similar activities in HFFF2 and Vero cells, and indeed the greatest degree of stimulation occurred in 3T3 cells for both viruses.

**Figure 2:**
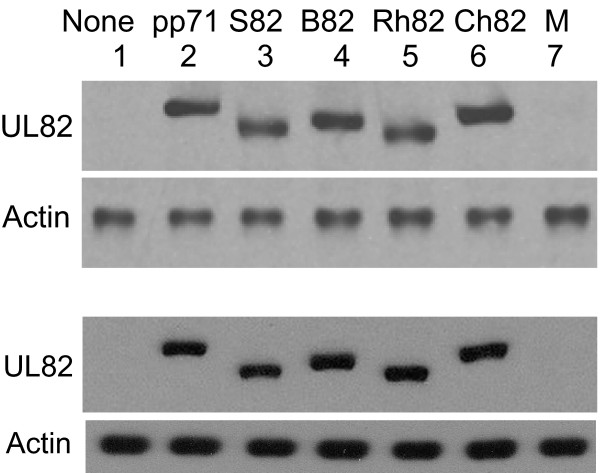
**Protein expression by *in*1312-based recombinants**. Monolayers of U373 cells were infected with 1 pfu/cell of *in*1374-based recombinants that expressed YFP-tagged UL82 homologs and maintained at 38.5°C for 10 h. At this time, extracts were analysed for protein levels, using anti-GFP or anti-actin antibodies as probes. Monolayers were infected with *in*1374 (lane 1), *in*1310 (lane 2), *in*0150 (lane 3), *in*0145 (lane 4), *in*0144 (lane 5), *in*0146 (lane 6) or mock infected (lane 7). Extracts were also analysed for β-galactosidase activity, as described in table 1.

The experimental approach described above, which relies on dual infection of cells, demonstrates that S82 activated transcription with an activity comparable to that of pp71. However, the use of sequential infections in the assay may compromise detection of small differences in activity. To examine more stringently the activities pp71 and S82, and to include comparisons with other UL82-derived proteins, YFP-tagged ORFs of pp71, S82, Ch82, B82 or Rh82 were cloned into the TK region of the HSV-1 mutant *in*1374, to produce recombinant viruses that express both UL82 homolog and β-galactosidase (Table 1). Infection with these mutants was more appropriate for investigating relatively small differences in activity of the UL82 homologs, since the protein and reporter sequences were delivered to cells on the same genome. The results reported here, using YFP-tagged proteins, have been reproduced with myc-tagged versions (not shown), confirming, as shown previously, that the YFP moiety does not affect the functional properties of the proteins [21,24,33]. The functional activities of the proteins were determined by infecting U373 monolayers at moi 1 or HFFF2 at moi 5 and incubation at 38.5°C for 10 h, followed by assay of β-galactosidase and analysis of the amounts of YFP-tagged UL82 homolog synthesized. Preliminary experiments demonstrated that β-galactosidase production was proportional to input virus up to moi 5 (results not shown). Expression of the UL82 homologs was approximately equivalent for the five recombinants in both cell types (Fig. 2). Expression of β-galactosidase, however, was 1.5–2.5 times greater in cultures expressing S82, B82 and Rh82 than in those expressing pp71 or Ch82 (Table 3). Therefore, all primate UL82 homologs activated gene expression, and those derived from SCMV, BCMV and RhCMV were more active when introduced into cells by infection with an *in*1374-derived recombinant.

**Table 3:** Expression of β-galactosidase in cells expressing UL82 homologs^1^

Virus	Protein expressed	β-galactosidase activity	
		U373	HFFF2
*in*1310	YFPpp71	181 (20)	240 (21)
*in*0150	YFPS82	400 (20)	450 (73)
*in*0145	YFPB82	497 (0)	521 (117)
*in*0144	YFPRh82	353 (15	382 (94)
*in*0146	YFPCh82	210 (10)	243 (16)
*in*1374	None	0 (13)	35 (2)

1. Monolayers of U373 cells or HFFF2 cells were infected with 1 pfu/cell (U373) or 5 pfu/cell (HFFF2) of *in*1374-based recombinants that expressed YFP-tagged UL82 homologs and maintained at 38.5°C for 10 h. At this time, extracts were prepared and assayed for β-galactosidase activity. Extracts were subsequently analyzed for protein production, as shown in figure 2. Background values (from mock infected cultures) were subtracted. The data presented here and in figure 2 are from the same lysates of a single experiment, and are representative of three independent experiments. The β-galactosidase activity from mock infected cells was subtracted from all values. The value in brackets is the maximum deviation from the mean of duplicate (U373) or triplicate (HFFF2) determinations.

**Figure 3:**
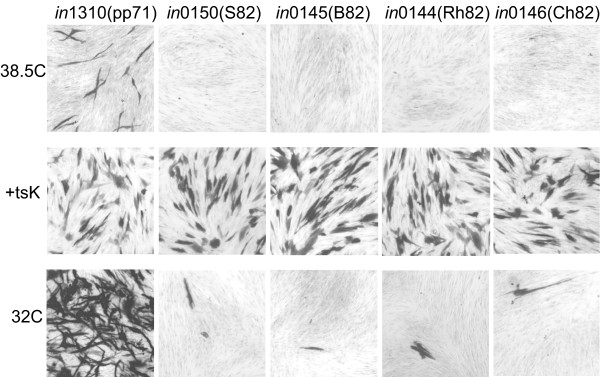
**Long term expression of β-galactosidase**. Monolayers of HFFF2 cells were infected with *in*1374-based recombinants (moi 3) and incubated at 38.5°C for 10 days in medium containing 2% fetal calf serum. Monolayers were stained for β-galactosidase activity on day 10 (top row), after superinfection with *ts*K on day 10 for 16 h (middle row), or after downshift to 32°C on day 10 and futher incubation for 4 days in medium containing 2% human serum (bottom row).

We investigated whether the UL82 homologs shared with pp71 the property of directing gene expression for extended periods after infection of human fibroblasts. Cultures of HFFF2 cells were infected with *in*1374-based recombinants at moi 3 and maintained at 38.5°C for 10 days. After this time, cultures were treated in three ways, following the protocol described previously [24]. For one set of cultures, histochemical staining for β-galactosidase was employed as a direct measure of continued gene expression mediated by the UL82 homolog. A second set was superinfected overnight with HSV-1 *ts*K. This mutant expresses the IE protein ICP0 and therefore reactivates β-galactosidase expression from cells harboring a quiescent genome, thereby defining the proportion of cells containing one or more quiescent genomes. The final set was transferred to 32°C for 4 days, with human serum in the culture medium, to provide a sensitive test for the presence of genomes that were capable of replication when the temperature sensitive defect of ICP4 was reversed. Cultures infected with *in*1310, encoding YFPpp71, contained β-galactosidase expressing cells after incubation at 38.5°C for 10 days but positive cells were not detected in cultures infected with recombinants expressing S82, B82, Ch82 or Rh82 (Fig. 3, top row), even though superinfection with *ts*K resulted in equivalent β-galactosidase expression in all cultures (Fig. 3, middle row). Upon downshift to 32°C, extensive replication, demonstrated by widespread plaque formation, was observed in *in*1310-infected cultures, but those infected with viruses encoding the other primate UL82 homologs contained no more than 15 β-galactosidase positive cells, or small clusters, per monolayer (Fig. 3, bottom row). These numbers were only marginally greater than those observed in cultures infected with the parental virus *in*1374 (not shown). Therefore, of the UL82 homologs tested, none exhibited the property of preventing or reversing the repression of HSV-1 genomes during long term incubation of HFFF2 cultures in the manner observed for pp71.

**Figure 4:**
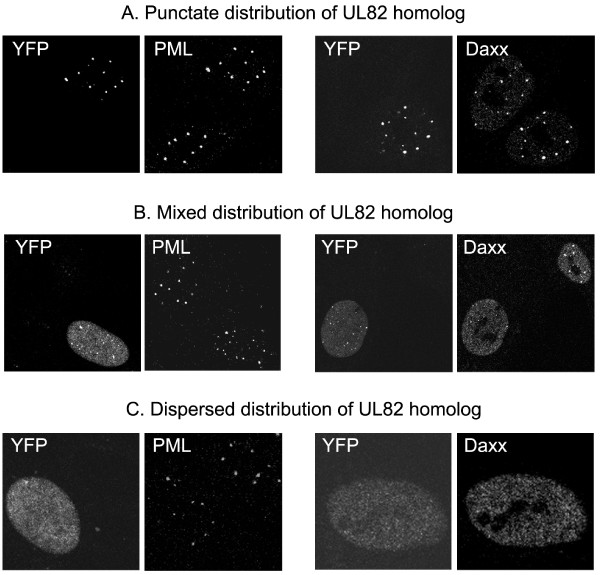
**Distribution patterns of UL82 homologs**. Monolayers of HFFF2 cells were infected with *in*1374 derivatives expressing YFP-tagged homologs and analysed by immunofluorescence at 3 h or 7 h post infection at 38.5°C. Representative images are presented to demonstrate the three distribution patterns that were scored and quantified to produce the data presented in table 4.

Interaction with the cell protein Daxx is important for the role of pp71 in activation of gene expression. The intranuclear localization of the UL82 homologs was investigated by immunofluorescence after infection of HFFF2 cells at moi 0.1 with the *in*1374-based recombinants that expressed YFP-tagged UL82 homologs. Monolayers were fixed and co-stained for Daxx at 3 h and 7 h post infection (pi). The patterns of YFP fluorescence were classed as punctate (in which the signal was exclusively localized to ND10), dispersed (no distinct foci) or mixed (punctate superimposed on a background of dispersed signal). Examples of these patterns are shown in figure 4, and it is noteworthy that the distribution of Daxx coincided with that of the YFP signal whereas the major ND10 component, PML, retained a punctate distribution even when the YFP and Daxx signals were dispersed. The effect of the UL82 homologs was therefore to alter the intranuclear localisation of Daxx rather than to disrupt ND10 structures totally. The distributions were quantified by counting several fields from at least two coverslips (Table 4). As described previously [21], at 3 h after infection with *in*1310 YFPpp71 colocalized with Daxx at ND10 in virtually all positive cells. At 7 h pi, again most cells showed a punctate distribution of YFPpp71, although those with greater amounts of protein exhibited a mixed pattern and in a small number YFPpp71 was dispersed (Table 4). The most obvious finding was that S82 exhibited a localization pattern very different from that of pp71. Even by 3 h pi, S82 was predominantly in a dispersed or mixed distribution with virtually no nuclei containing punctate signal. By 7 h pi, 86% of S82-containing nuclei exhibited a dispersed distribution of the protein whereas, by contrast, only 1% of pp71-positive profiles were dispersed. The localizations of the other three homologs, B82, Rh82 and Ch82, were intermediate between those of pp71 and S82, with mainly punctate appearance at 3 h pi but increased dispersed or mixed distribution at 7 h pi. Surprisingly, pp71 was more similar to Rh82 than to Ch82 in this respect.

**Table 4:** Intranuclear distributions of UL82 homologs

Protein	Distribution 3 h pi (% total)			Distribution 7 h pi (% total)		
	Punctate	Mixed	Dispersed	Punctate	Mixed	Dispersed
YFPpp71	97	3	0	70	29	1
YFPS82	0	48	52	0	16	84
YFPB82	84	16	0	26	39	35
YFPRh82	84	15	1	64	28	8
YFPCh82	90	10	0	38	40	22

HFFF2 monolayers were infected with *in*1374-based recombinants that expressed UL82 homologs, and maintained at 38.5°C. At 3 h pi and 7 h pi, cells were fixed and prepared for immunofluorescence. Distributions of proteins, as shown in figure 4, were scored for between 95 and 151 YFP-positive nuclei.

**Figure 5:**
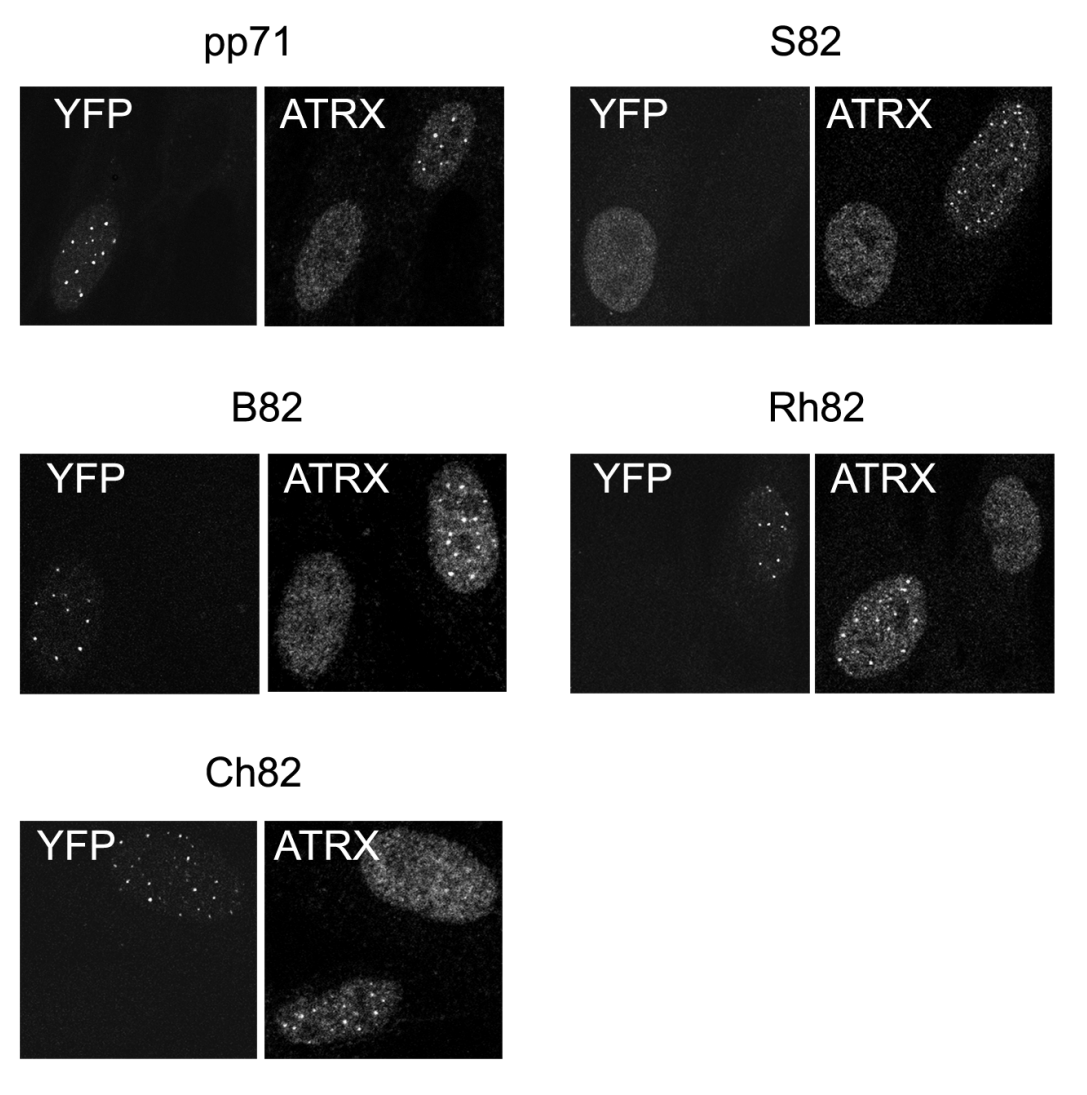
**Distribution patterns of UL82 homologs and ATRX**. Monolayers of HFFF2 cells were infected with *in*1374 derivatives expressing YFP-tagged homologs and analysed by immunofluorescence at 3 h post infection at 38.5°C.

It was reported previously that infection with *in*1316 resulted in the dispersal of ATRX at 3 h pi, a time at which YFPpp71 and Daxx remained at ND10 in a punctate distribution [21]. To investigate whether the UL82 homologs also promoted the early dispersal of ATRX, nuclei were examined at 3 h pi and 25 YFP positives scored for ATRX distribution. It was clear that expression of all UL82 homologs resulted in dispersal of ATRX even though the YFP signal was punctate (Fig. 5), and this was confirmed by quantification (Table 5). The homologs are therefore similar to pp71 in their abilities to promote the early dispersal of ATRX from ND10.

**Table 5:** Intranuclear distributions of UL82 homologs and ATRX at 3 h pi

Protein	Distribution of YFP (number)			Distribution of ATRX (number)		
	Punctate	Mixed	Dispersed	Punctate	Mixed	Dispersed
YFPpp71	23	2	0	0	4	21
YFPS82	0	11	14	0	3	22
YFPB82	22	3	0	1	2	22
YFPRh82	21	4	0	0	2	23
YFPCh82	20	5	0	1	7	17

HFFF2 monolayers were infected with *in*1374-based recombinants that expressed UL82 homologs, and maintained at 38.5°C. At 3 h pi, cells were fixed and prepared for immunofluorescence. Distributions of proteins, as shown in figure 4, were scored for 25 YFP-positive nuclei.

## Discussion

The cytomegaloviruses are overtly similar in terms of biological properties and coding potential, but differences can be discerned when the viruses are considered in greater detail. One obvious variation is in overall genome structures, since HCMV and ChCMV have inverted repeat sequences that mediate inversion of the unique regions [34,35], whereas RhCMV, like MCMV, lacks internal repeats and does not invert [36-38]. In terms of gene regulation HCMV, SCMV and MCMV each utilize a potent, complex enhancer region for transcriptional control of the major IE region, but there are few strong sequence similarities between these regulatory elements [39-41]. Previous studies demonstrated differences between HCMV and SCMV IE transcription, with certain cell types that restricted HCMV IE transcription nonetheless permitting full expression of the SCMV IE region [42,43]. The findings described here suggest that these differences reflect the nature of the IE enhancer regions rather than a cell-type specific actvity of the relevant UL82 homolog.

The results presented here demonstrate that four non-human primate UL82 homologs stimulate transcription in a manner that is generally similar to that of pp71. The strong sequence homology suggested that this would be the case, but it should be noted that the predicted MCMV UL82 homolog does not appear to activate gene expression whereas GPCMV UL82 homolog, which lacks many features conserved in the primate proteins, has functional activity [25-28].

Insertion of ORFs into the HSV-1 recombinants *in*1312 and *in*1374 provided a useful system for investigating the activities of UL82 homologs, yielding comparative information that would be difficult to obtain by studying the parental cytomegaloviruses themselves. At a practical level, infection with *in*1312 derivatives permits delivery of transgenes into cells at constant, relatively low copy numbers whereas transfection results in heterogeneity in the amount of DNA delivered to individual cells and hence large variations in gene expression at the individual cell level. It should be noted, however, that the basic properties of the UL82 homologs reported here were reproduced when plasmids expressing them were introduced into cells by transfection, and that pp71 mutants with known inactivating mutations, such as deletion of the Daxx interacting domains [4,7], were non-functional when expressed from *in*1374-based recombinants (results not shown). An additional advantage of using *in*1374-based recombinants is that the reporter sequences (encoding β-galactosidase) are present in a herpesvirus genome and hence should, to some extent, represent the normal environment of UL82 target promoters.

It was surprising to find that only pp71 was able to direct long term gene expression, in view of the fact that all homologs were functional in the short term assay. This result suggests that the ability to direct long term expression is a specific feature of pp71 that is distinct from its ability to activate IE transcription. Indeed, this conclusion is supported by our the observation that, to date, six pp71/Ch82 hybrid proteins that we have expressed from plasmid constructs actively stimulated IE gene expression but failed to direct long term expression (results not shown). The most likely explanation for these findings is that mediating long term expression requires interactions between different domains of the pp71 molecule, and that equivalent regions of Ch82 are incompatible. At present, there is no obvious biological consequence of this unique property of pp71.

The most notable finding from an analysis of intranuclear distributions of the UL82 homologs was the distinct behaviour of S82, which exhibited a dispersed distribution and mediated the dissociation of Daxx from ND10 at earlier times than the other proteins. The difference in intranuclear localisation between pp71 and S82 was also observed after infection of African green monkey fibroblasts with *in*1310 and *in*0150, demonstrating that cell type is not a crucial variable (results not shown). Surprisingly, the more rapid dispersal of Daxx from ND10 was not reflected in greater functional activity of S82; although S82 was approximately twice as active as pp71 in the expression assay, it was similar to B82 and Rh82, which were predominantly ND10-associated at 3 h and, for Rh82, 7 h pi. The S82 protein was not produced more rapidly than pp71 (results not shown). All homologs promoted the rapid dissociation of ATRX from ND10, irrespective of the effects on the distribution of Daxx, supporting the recent report that the most important effect of pp71 at early times of infection may be dissociation of cellular repressor proteins, such as ATRX, from ND10 rather than direct effects on Daxx itself [21].

## Conclusion

The results presented here reveal surprising dissimilarities in the properties of primate cytomegalovirus UL82 homologs, despite the fact that each of the proteins is a potent transactivator of IE gene expression. The close relatives, pp71 and Ch82, are slightly lower in transactivation activity than the other three, but Ch82 lacks the ability to direct long term expression and is sequestered at ND10 less thoroughly than pp71. The functional heterogeneity extends through the UL82 proteins studied here, since S82 is similar in functional activity to B82 and Rh82 but strikingly different in its intranuclear localization. Further studies to determine the significance of the differences between UL82 homologs may require investigation of the cytomegaloviruses themselves. It may also be possible to construct recombinant cytomegaloviruses containing heterologous or hybrid UL82-encoded proteins, provided there are no problems with incompatibilities for incorporation of the proteins into the virus particle.

## Materials and methods

### Identification and cloning of S82, the SCMV UL82 homolog

DNA from SCMV (strain Colburn) was purified from virions released into culture medium and digested with HindIII and EcoRI. The mixture was cloned into pUC9 that had been digested with HindIII and EcoRI. Plasmids, from colonies picked at random, were sequenced from their ends, and an isolate containing a 1 kilobase pair (kbp) insert homologous to HCMV UL76 was selected. The insert was used as a probe for colony hybridization to detect a plasmid containing a 7 kbp HindIII/NheI fragment of which one terminus was homologous to HCMV UL80. A fragment from this plasmid was used as probe to identify a 5 kbp EcoR1/AgeI fragment with one end homologous to the coding region of HCMV UL83 and the other homologous to the C-terminus and termination codon of HCMV UL82, plus 150 bp of downstream sequence. It was concluded that the EcoRI/AgeI fragment contained the entire coding region of the SCMV UL82 homolog (named S82). Further analysis determined that the S82 ORF was contained entirely within a 1.9 kbp region defined by NotI and SacII sites. This fragment was cloned between the NotI and SacII sites of pBluescript SKII and the nucleotide sequence determined. The S82 ORF was isolated by polymerase chain reaction using Vent polymerase and primers 5'-GGACGGCTAAGCTTGGATGGATCGCCCTCCCGAGG and 5'-GGGCCGAATTCCAGGATTCGAGGTCTCGCAGTGG, thereby introducing HindIII and EcoRI sites flanking the 5' and 3' termini of the ORF, respectively. The HindIII/EcoRI fragment was cloned between the HindIII and EcoRI sites of pEYFP-C1 to produce plasmid pYFPS82, and the S82 termination codon was regenerated by site-directed mutagenesis to yield pYFPS82T11.

### Derivation of a phylogenetic tree

Amino acid sequences were aligned using CLUSTAL W [44], and locations in the alignment with a gapping character in any sequence were removed. A phylogenetic tree was constructed from the alignment by the neighbor-joining method, using programs in the PHYLIP package [45].

### Viruses

The HSV-1 mutant *in*1312 has been described previously [46]. Derivatives *in*1374 [24] and *in*1382 [3], contain HCMV IE-lacZ inserted at the UL43 and TK locus, respectively. Derivative *in*1357 has an insertion consisting of *lacZ *controled by the SCMV IE promoter at the TK locus [5], and *in*1383 has the HSV-1 ICP0 promoter controling *lacZ*, inserted at the TK locus of *in*1312 [46]. Mutant *in*1372 expresses Cre recombinase in the *in*1312 background [47].

### Construction of HSV-1-derived recombinants that express UL82 homologs

To produce HSV-1 recombinants, UL82 homologs were tagged at the N-terminus with YFP, and the resulting hybrid ORFs were cloned into pCP1802, a transfer vector in which sequences can be inserted, under the control of the HCMV MIEP and simian virus 40 termination signals, into the HSV-1 thymidine kinase (TK) coding region [3]. The resulting plasmids were linearized and cotransfected with DNA from *in*1312 or *in*1374. Selection of TK-deficient mutants and analysis by Southern hybridization were carried out as described previously [48]. The methods used for the individual UL82 ORFs are as follows:

1. The construction of pYFPpp71, pMJ129 (YFPpp71 cloned into the HpaI site of pCP1802) and recombinant viruses that express YFPpp71 (*in*1316, derived from *in*1312, and *in*1310, derived from *in*1374) have been described previously [5,24].

2. The B82 ORF was excised as a SalI/NotI fragment from a plasmid containing the entire ORF, and cloned between SalI and NotI sites of pYFPpp71 to replace the pp71 ORF with that of B82. YFP-tagged B82 was excised as an AgeI/NotI fragment, the ends filled in and cloned into the HpaI site of pCP1802 to yield plasmid pMJ187.

3. To clone the Rh82 ORF, pYFPpp71 was first modified by insertion of an oligonucleotide duplex (top strand 5'-CCGGAATGGATCGCCCTCCCGAGGAAGAAGAAGAGCCCAGGCCGTCTACCTCTCGAG) between the BspEI and EcoRI sites, thereby replacing the pp71 ORF with the N-terminal 17 amino acids of Rh82. The remainder of the Rh82 ORF was cloned as an XhoI/DraI fragment from a plasmid containing genomic DNA encompassing the Rh82 ORF, between XhoI and SmaI sites of the modified plasmid. The YFPRh82 coding region was excised as an AgeI/NotI fragment, end-filled and cloned into the HpaI site of pCP1802 to yield pMJ185.

4. The Ch82 ORF was amplified from ChCMV DNA (kindly provided by A. Davison, MRC Virology Unit), using primers 5'-GTCTCGATCTCCTTCGCC and 5'-CCATGATCACAGCAGTGG, cloned into pGEMT-EASY to yield pMJ162. The insert of this plasmid was sequenced to confirm that there were no changes from the published data [35]. Plasmid pYFPpp71 was modified by insertion of an oligonucleotide duplex (top strand 5'-TCGAGCTATGTCTCGATCTCCTTCGCCCGGGGAAGGGCCCGAACAAG) between XhoI and BamHI sites, thereby replacing the pp71 ORF with the N-terminal 11 amino acids of Ch82. The remainder of the Ch82 ORF was excised from pMJ162 as an ApaI/SpeI fragment and cloned between ApaI and SpeI sites of the modified plasmid, to yield pTC2. The YFPCh82 ORF was excised from pTC2 by cleavage with AgeI and SpeI, end-filled and cloned into the HpaI site of pCP1802 to yield pTC9.

5. To clone YFPS82, an AgeI/BamHI fragment from pYFPS82T11 was filled in and cloned into the HpaI site of pCP1802 to yield pJS998.

To produce recombinant viruses, ScaI-linearized pMJ135, pMJ137, pTC9 or pJS998 were cotransfected with *in*1312 or *in*1374 DNA. Acycloguanosine resistant viruses were plaque purified and analysed by Southern hybridization for the presence of the transgene in the TK locus. Isolates lacking detectable parental sequences on long autoradiographic exposures were propagated in BHK cells and titrated at 32°C on U2OS monolayers in the presence of 3 mM hexamethylene bisacetamide [49]. The content of functional genomes was assessed by infection of U373 and HFFF2 cells at low moi and coinfection overnight at 38.5°C with the HSV-1 mutant *ts*K, which provides the IE protein ICP0 and overcomes repression of genomes, and counting of β-galactosidase positive cells. The 'functional genome content' determined by this method agreed closely with the titres obtained on U2OS monolayers, confirming that the preparations contained comparable genome contents. A summary of the mutants used in this study is presented in table 1.

### Cells

Human fetal foreskin fibroblasts (HFFF2), African green monkey (*Cercopithecus aethiops*) Vero cells, mouse Swiss 3T3 cells, human glioblastoma U373-MG cells and human osteosarcoma U2OS cells were propagated in Dulbecco's modified Eagle medium supplemented with nonessential amino acids, 5% fetal calf serum, 5% newborn calf serum, 100 units of penicillin and 100 μg of streptomycin per milliliter. BHK cells were propagated in Glasgow modified Eagle medium supplemented with 10% newborn calf serum, 10% tryptose phosphate broth, 100 units of penicillin and 100 μg of streptomycin per milliliter.

### Assays for effects on gene expression

Two assays were used for short term assay for stimulation of gene expression. In the first of these, monolayers of HFFF2, Vero or 3T3 cells were mock infected or infected (moi 2) with an *in*1312 derivative expressing a UL82 homolog or control virus *in*1372, an *in*1312 derivative that expresses Cre recombinase [47], and incubated at 38.5°C for 3 h. Cultures were superinfected with a second *in*1312 derivative (moi 0.5) containing *lacZ *controlled by various promoter elements. Incubation was continued at 38.5°C for 5 h, cells were harvested and β-galactosidase assayed using methylumbelliferyl-β-D-galactoside as substrate, as described previously [50]. Alternatively, monolayers of U373 or HFFF2 cells were infected with an *in*1374 derivative that expressed a UL82 homolog (and also contained *lacZ *coding sequences). At 10 h post infection, extracts were prepared and assayed for β-galactosidase activity, as described above, and analysed for protein expression by western blotting using rabbit anti-green fluorescent protein antibody (Abcam) and mouse anti-actin antibody (Sigma-Aldrich) as probes, as described previously [5]. Long term expression was analysed after infection of HFFF2 monolayers with *in*1374 derivatives expressing UL82 homologs (moi 3), as described previously [24]. Infected monolayers were maintained at 38.5°C for 10 days, at which time some were maintained at 38.5°C, some were transferred to 32°C for 4 days in medium supplemented with 2% human serum, and others were infected with the HSV-1 ICP4 mutant *ts*K and maintained overnight at 38.5°C. Monolayers were fixed and stained for β-galactosidase expression as described previously [48].

### Immunofluorescence

Coverslips were prepared and analysed by confocal microscopy as described previously [51]. Primary antibodies were rabbit anti-Daxx (Upstate), rabbit anti-ATRX (Santa Cruz) or mouse anti-PML, clone PG-M3 (Santa Cruz). Secondary antibodies were Cy3- or Cy5-conjugates (GE Healthcare).

## Competing interests

The authors declare that they have no competing interests.

## Authors' contributions

IPN isolated the S82 ORF and carried out initial cloning and functional analysis. JSS, TNC and MJN contributed to the cloning and functional analyses of UL82 homologs. ELB and PAB provided plasmids containing the B82 and Rh82 ORFs, respectively. CMP carried out functional analyses and immunoflourescence, and was responsible for preparation of the manuscript.
